# Co-culture of Mouse Embryonic Stem Cells with Sertoli Cells Promote *in vitro* Generation of Germ Cells

**Published:** 2013-06

**Authors:** Mohammad Miryounesi, Karim Nayernia, Mahdi Dianatpour, Fatemeh Mansouri, Mohammad Hossein Modarressi

**Affiliations:** 1 Department of Medical Genetics, School of Medicine, Tehran University of Medical Sciences, Tehran, Iran; 2 GENEOCELL, Advanced Molecular & Cellular Technologies, Montreal, Canada; 3 Department of Medical Genetics, School of Medicine, Shiraz University of Medical Sciences, Shiraz, Iran

**Keywords:** Co-culture, Differentiation, Embryonic stem cell, *In vitro* derived germ cells, Sertoli cell

## Abstract

***Objective(s):*** Sertoli cells support *in vivo* germ cell production; but, its exact mechanism has not been well understood. The present study was designed to analyze the effect of Sertoli cells in differentiation of mouse embryonic stem cells (mESCs) to germ cells.

***Materials and Methods:*** A fusion construct composed of a *Stra*8 gene promoter and the coding region of enhanced green fluorescence protein was produced to select differentiated mESCs. To analyze sertoli cells’ effect in differentiation process, mESCs were separated into two groups: the first group was cultured on gelatin with retinoic acid treatment and the second group was co-cultured with sertoli cell feeder without retinoic acid induction. Expressions of pre-meiotic (*Stra*8), meiotic (*Dazl* and *Sycp*3) and post-meiotic (*Prm*1) genes were evaluated at different differentiation stages (+7, +12 and +18 days of culture).

***Results:*** In the first group, expressions of meiotic and post-meiotic genes started 12 and 18 days after induction with retinoic acid, respectively. In the second group, 7 days after co-culturing with Sertoli cells, expression of meiotic and post-meiotic genes was observed.

***Conclusion:*** These results show that differentiation process to germ cells is supported by Sertoli cells. Our findings provide a novel effective approach for generation of germ cell *in vitro* and studying the interaction of germ cells with their niche.

## Introduction

An estimated 10 to 15 percent of couples worldwide have fertility problems, half of whom being infertile men (1). The causes of male infertility are numerous. One of the untreatable causes is sertoli-cell-only (SCO) syndrome which is characterized by azoospermia and lack of germ cells in testis biopsy (2). Recent studies have shown that mouse embryonic stem cells (mESCs) are able to give rise to primordial germ cells (PGCs) and sperm-like cells *in* *vitro* (3). mESCs are derived from inner cell mass and have two properties: self renewal and pluripotency (4). The ability to generate germ cells from mESCs provides a powerful *in vitro* model to study germ cell development and offers new therapeutic approaches to infertility (5).

Spermatogenesis is a complex process in which undifferentiated germ cells undergoes successive mitotic and meiotic divisions and a morphological reorganization, in order to produce a cell with capability of fertilizing an oocyte. The process of mammalian spermatogenesis can be divided into two phases: 1) prenatal phase consists of the migration of primordial germ cells (PGC) from yolk sac to gonadal ridges and testicular organogenesis and 2) a postnatal phase in which spermatogonial stem cells differentiate into mature spermatozoa. In prenatal phase, PGCs (spermatogonial precursors) and Sertoli cell precursors aggregate and form the testicular cords (6).

Spermatogenesis is influenced by many factors, such as hormones (endocrine and paracrine), cell-cell interaction, surrounding microenvironment and many other factors. One of the most important factors is Sertoli cells whose role in supporting spermatogonial stem cells has been proved. It has been shown that Sertoli-germ cell communication guarantees the production of healthy sperms (7). In a recent study, it has been shown that conditioned medium from sertoli cell contain some factors that could induce *in vitro* germ cell differentiation (8). 

Deleted in Azoospermia-Like (Dazl) gene regulates germ cell development. Dazl is expressed during spermatogenesis in spermatogonia and primary spermatocytes and it seems to play a key role in meiosis initiation (5). Synaptonemal complex (SC) is a meiotic-specific structure. There are three SC component proteins in mammals (SCP1, SCP2 and SCP3). SCP3 (known as Sycp3) is supposed to constitute the core of these proteins (9). Finally, *Prm*1 which codes for protamines, is a post-meiotic gene with specific expression in haploid round spermatids (10).

The purpose of the present study was to compare the process of* in* *vitro* differentiation of mESCs to germ cell in two different conditions: regular culture system and co-culture on Sertoli cells as feeder layer. 

## Materials and Methods

***Cell culture***

Mouse embryonic stem cell line C57BL6 with normal male (XY) karyotype (Invitrogen) was cultured in an undifferentiated state on a feeder layer of mitomycin C-inactivated mouse embryonic fibroblasts (11). Culture medium composed of Knockout Dulbecco’s modified Eagle’s medium (Knockout DMEM, GIBCO-BRL) was supplemented with 12.5% (v/v) ES qualified FBS (GIBCO-BRL), 2 mmol L-Glutamine (GIBCO-BRL), 1X nonessential amino acids (NEAA; GIBCO-BRL), 50 µgml^-1^ penicillin and streptomycin, 50 µmol β-mercaptoethanol and 10³ unit ml^-1^ LIF (Milipore). Mouse Sertoli cell line was then cultured in the medium composed of Ham'S F12 and Dulbecco’s modified Eagle’s medium (DMEM), 2.5 % (v/v) Fetal bovine serum (FBS, GIBCO-BRL) and 5% (v/v) Horse serum (Sigma).

***Construction of germ cell specific reporter gene and recombinant ES cell***

A segment of *Stra*8 (Stimulated by Retinoic Acid 8) gene (-1400/+7) was amplified from genomic DNA and inserted in the SacI/HindIII site of modified pEGFP-1 vector, where neomycin-resistance cassette was replaced with puromycin-resistance gene (12). ES cells were trypsinized and around 7×10^6^ cells were prepared for performing electroporation. Linearized vector (35 µg) was electroporated into ES cells. Electroporation was performed on Bio-Rad GenePulser device at 250 V and 500 µF. After electroporation, mESCs were transferred into six wells plate and cultured for 72 hr without antibiotic. Puromycin (final concentration 1 µg ml^-1^) was added to the medium and cells were cultured in the presence of puromycin for three weeks. Subsequently, puromycin resistant colonies were selected. DNA extraction was performed from these colonies tested for presence of *Stra*8/EGFP vector by PCR. Positive colonies were grown in a complete medium with LIF for a month.

***Derivation of germ cells from mESCs***

**Figure 1:**
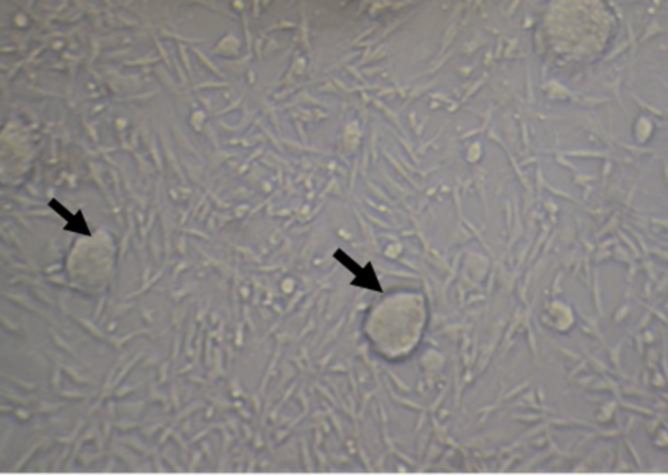
Co-culture of mESCs (arrows) colonies and Sertoli cells

Retinoic acid (RA) was added to the medium at a final concentration of 10ˉ^5^ mol for 72 hr to induce differentiation. GFP-positive cells were then selected using fluorescence-activated cell sorting (FACS). Cells were trypsinized and after pipetting up and down few times filtered through a 40 μm strainer (Falcon) to create single-cell suspensions. Sorting was carried out with 3×10^6^ cells using BD FACS Aria II flow cytometer (13). These cells were cultured on an MEF feeder under non-induced condition for two weeks. Resulting colonies were divided into two groups for comparing the process of differentiation in different conditions. Group 1 was cultured for 18 days on gelatin with RA treatment (final concentration 10^-8^ mol) and group 2 was co-cultured on mouse Sertoli cell line (TM4) without RA treatment (Figure 1). The culture media of both groups was the same (except for RA). 

***RNA extraction, cDNA synthesis and RT-PCR***

Total RNA was extracted using TRIzol reagent (Invitrogen) according to the manufacturer’s protocol. RNA concentration was measured with Nano Drop 1000 spectrophotometer (Thermo Fisher Scientific). The extracted RNA (2 µg) was employed for cDNA synthesis using M-MLV reverse transcriptase (Fermentase) with random hexamer and oligo dT primer together. RT-PCR was performed using a specific primer for EGFP, *Oct*4, *Stra*8, *Sycp*3, *Dazl* and *Prm*1genes (Table 1). 

## Results

***Establishment of spermatogonial stem cell (SSC) like cells***

In order to isolate differentiated stem cells, a specific reporter construct consisting of a germ line-specific segment of *Stra*8 gene promoter and the coding region of enhanced green fluorescence (EGFP) protein were used. The use of this 1.4 Kb *Stra*8 promoter caused testis-specific expression of GFP. After selection with puromycin, ES colonies were chosen and PCR with GFP specific primers was *Dazl*, *Sycp*3 and *Prm*1 were not detected in induced and non-induced cells. Moreover, *Stra*8 is a molecular marker for spermatogonial stem cells (13); therefore it was concluded that induced cells were spermatogonial stem cell (SSC)-like cells. 

**Figure 2a:**
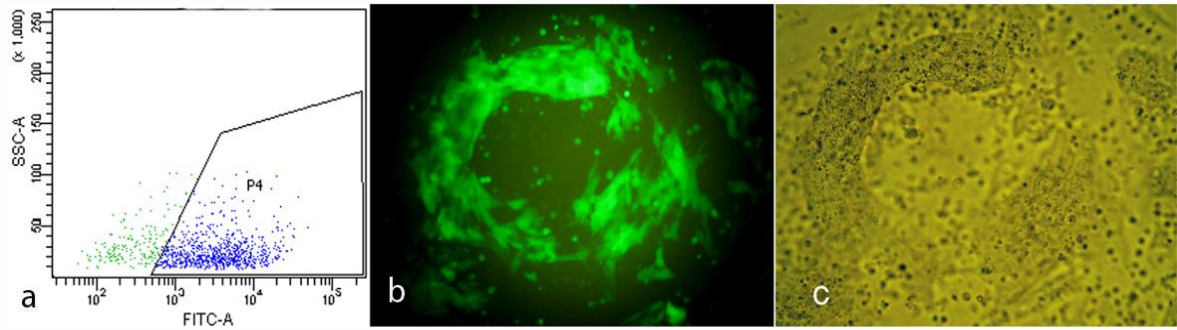
Diagram of GFP expressed mESCs sorted by FACS. b and c: Sorted cells showed high expression of GFP

***Differentiation of SSC-like cells in different conditions***

**Figure 3:**
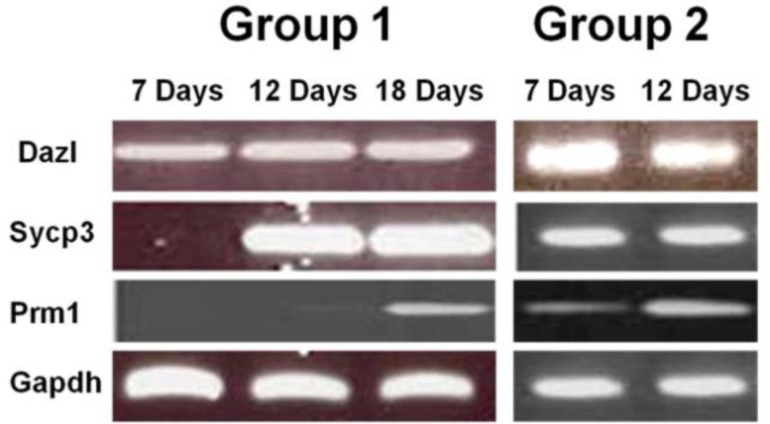
Expression of meiotic (*Sycp*3 and *Dazl*) and postmeiotic (*Prm*1) genes in two groups of culture: Group 1: culture of mESCs on gelatin with RA treatment,Group 2: co-cultured of mESCs with Sertoli cell feeder without RA induction

In this study, SSC-like cells were cultured in two groups and mRNA of these cells was extracted in periods of 7, 12 and 18 days of culture (Figure 3). These samples were analyzed for pre-meiotic (*Oct*4 and *Stra*8), meiotic (*Sycp*3 and *Dazl*), and post- meiotic (*Prm*1) genes expression. In both groups, expression of *Oct*4, *Stra*8, and *Dazl* were observed on expression was started on the 7^th^ day in the second group. There was no expression of *Prm*1 until the 7^th^ day of cell culture. In the first group, Sycp3 was expressed on the 12^th^ day of culture, though the 18^th ^ day in the first group. However, the expression of *Prm*1 started on the 7^th^ day of culture in group 2 (Table 2). As a control, the expression of these genes was negative in the TM4 Sertoli cell line (data not shown). 

## Discussion

This study provides an *in vitro* model of spermatogenesis in which the effects of cell-cell interaction and microenvironment may be examined. 

During transition of mammalian germ cells from mitosis to meiosis *Stra*8 gene is expressed (14). A vector composed of promoter region of *Stra*8 gene and GFP coding sequence was used in this study to select stable transfected ES cells that entered meiosis. We observed that if we GFP-positive cells were not separated, which entered meiosis stage in the cell culture; differentiation process could be suppressed by neighboring undifferentiated cell populations. This finding has also been observed previously (15) and that is why FACS was used for cellular separation.

*Dazl* is a testis-specific gene with a critical role in the spermatogenesis. Recent studies have shown that *Dazl* is a key factor in meiosis initiation (16, 17). Deletion of *Dazl* gene in knockout mice, results in meiosis arrest which may reduce the expression of some specific genes involved in sperm production (5). In this study, detection of *Dazl* expression showed that SSC-like cells has been entered meiosis in both groups (with and without Sertoli cells) after seven days of culture.* Sycp*3 is a meiotic specific gene responsible for the formation of synaptonemal complex. This complex has an important role in synapses formation, recombination and segregation of chromosomes during meiosis (9). 

**Table 1:** r sequence and expected size of PCR products of *Stra*8, *EGFP*, *Dazl*, *Sycp*3 and protamine-1 genes

Gene	Primer sequence	Product size (bp)
*Stra*8	F : ACAACCTAAGGAAGGCAGTTTAC R: TGACCTCCTCTAAGCTGTTGG	174
EGFP	F: GCACCATCTTCTTCAAGGACGAC R: TCTTTGCTCAGGGCGGACTG	270
*Dazl*	F: CAGGCATATCCTCCTTATCCAAG R: TGTATGCTTCGGTCCACAGAC	263
*Sycp*3	F: CCGGAGCCGCTGAGCAAACA R: CCAGTTCCCACTGCTGCAACAC	430
*Prm*1	F: CTCACAGGTTGGCTGGCTCGAC R: CGGCGACGGCAGCATCTTCG	192

**Table 2:** Expression pattern of* Oct4*, *Stra*8, *Dazl*, *Sycp*3 and *Prm*1 genes in two groups: Group 1: culture of mESCs on gelatin with RA treatment, Group 2: culture of mESCs on Sertoli cells without RA induction

		*Oct*4	*Stra*8	*Dazl*	*Sycp*3	*Prm*1
Undifferentiated mESCs		+	−	−	−	−
after 72 hr induction with RA		+	+	−	−	−
Group 1	Day 7	+	+	+	−	−
	Day 12	+	+	+	+	−
	Day 18	+	+	+	+	+
Group 2	Day 7	+	+	+	+	+

Histones were replaced by Protamin1, a post-meiotic gene, in sperm chromatin (15). The difference in expression pattern of *Sycp*3 and *Prm*1 genes in two groups may be due to the Sertoli cells role in the process of differentiation of mESCs to germ cells. 

Sertoli cells are located within the seminiferous tubules and support spermatogenesis through cell-cell contact (18). It has been shown that Sertoli cells are the main site of RA production in the testis. Sertoli cells provide RA for germ cells in two different ways: by direct delivery of RA to germ cells and delivery of retinol via membrane receptor, STAR6 (19). These findings support our results that Sertoli cells provide some necessary factors for *in vitro* differentiation of mESCs. Co-culture of ESC and Sertoli cells will promote differentiation process, number and diameter of colonies compared to the culture of ESC in gelatin. These results will be supported by further studies whit using the co-culture of extracted germ and Sertoli cells from human testis (20). 

## Conclusion

Differentiation is a complex process which requires many differentiating signals and Sertoli cells secret germ cell supporting factors such as RA. This model of *in vitro* germ cell production may open a new window to understand the effect of other factors on germ cell differentiation. 
